# Parietal Dysgraphia: Characterization of Abnormal Writing Stroke Sequences, Character Formation and Character Recall

**DOI:** 10.1155/2007/906417

**Published:** 2007-05-30

**Authors:** Yasuhisa Sakurai, Yoshinobu Onuma, Gaku Nakazawa, Yoshikazu Ugawa, Toshimitsu Momose, Shoji Tsuji, Toru Mannen

**Affiliations:** ^1^Department of NeurologyMitsui Memorial HospitalTokyoJapan; ^2^Department of NeurologyGraduate School of MedicineUniversity of TokyoTokyoJapan; ^3^Department of RadiologyGraduate School of MedicineUniversity of TokyoTokyoJapan

**Keywords:** Apraxic agraphia, parietal pure agraphia, intraparietal sulcus, limb apraxia, somatosensory area

## Abstract

*Objective:* To characterize various dysgraphic symptoms in parietal agraphia.

*Method:* We examined the writing impairments of four dysgraphia patients from parietal lobe lesions using a special writing test with 100 character kanji (Japanese morphograms) and their kana (Japanese phonetic writing) transcriptions, and related the test performance to a lesion site.

*Results:* Patients 1 and 2 had postcentral gyrus lesions and showed character distortion and tactile agnosia, with patient 1 also having limb apraxia. Patients 3 and 4 had superior parietal lobule lesions and features characteristic of apraxic agraphia (grapheme deformity and a writing stroke sequence disorder) and character imagery deficits (impaired character recall). Agraphia with impaired character recall and abnormal grapheme formation were more pronounced in patient 4, in whom the lesion extended to the inferior parietal, superior occipital and precuneus gyri.

*Conclusion:* The present findings and a review of the literature suggest that: (i) a postcentral gyrus lesion can yield graphemic distortion (somesthetic dysgraphia), (ii) abnormal grapheme formation and impaired character recall are associated with lesions surrounding the intraparietal sulcus, the symptom being more severe with the involvement of the inferior parietal, superior occipital and precuneus gyri, (iii) disordered writing stroke sequences are caused by a damaged anterior intraparietal area.

## 1. Introduction

Agraphia caused by lesions of the parietal lobe presents as three types with different anatomical substrates: alexia with agraphia from an angular gyrus lesion, pure agraphia from a superior parietal lobule lesion and agraphia with conduction aphasia [5,32].

Little is known about the clinical features of pure agraphia from a superior parietal lobule lesion [2,3]; the patients make phonemic paragraphia consisting of substitutions and omissions. A lesion in the superior parietal lobule [1] or surrounding the intraparietal sulcus [30] is known to cause apraxic agraphia, which is characterized by difficulty in forming graphemes. This condition produces inversions and distortions, despite normal sensorimotor function and relatively preserved oral spelling and typing using anagram letters [11]. Pure agraphia in general differs from apraxic agraphia in that patients usually make well-formed graphemes (written letters) [32]. One patient reported as having pure agraphia from a superior parietal lobule lesion [2] showed poorly formed graphemes and thus should be regarded as having apraxic agraphia. However, it remains unknown whether parietal pure agraphia and apraxic agraphia share the same lesion.

ISSN 0953-4180/07/$17.00 © 2007 – IOS Press and the authors. All rights reserved

The Japanese writing system uses two distinct scripts: kanji (morphograms or ideograms, originally adopted from Chinese characters) and kana (Japanese phonetic writing or syllabograms, originally taken from kanji characters). Some kanji characters with complex figures require many writing stroke sequences. Primary school students learn the order of kanji and kana character writing stroke sequences. Due to the use of these dual systems, agraphia in Japan presents itself in somewhat unique and pronounced ways. For example, (1) agraphia of kanji resulting from impaired character recall (lexical or orthographic agraphia) occurs in lesions of the posterior inferior temporal cortex [19,36, 41], angular gyrus [14,17,25,38], superior parietal lobule [17,23] and posterior middle frontal gyrus [35]; (2) paragraphia of kana (phonological agraphia) occurs in lesions of the supramarginal gyrus [37,43] and the posterior part of the middle and inferior frontal gyri [35]; and (3) Japanese apraxic agraphia patients with a superior parietal lobule lesion show disordered stroke sequences and distortions in writing kanji [14,29].

On the basis of the above findings (1) and (2), we previously hypothesized that visual images of kanji and kana words (and letters) are stored in the posterior inferior temporal cortex (and also the angular/lateral occipital gyri in case of kana characters) and travel via the angular gyrus and superior parietal lobule to the frontal motor and premotor areas (orthographic route, formerly "morphologic route") [35], whereas phonological information of words and letters goes from the primary auditory cortex and the posterior superior temporal gyrus to the angular and supramarginal gyri and joins the arcuate fasciculus to travel to the frontal motor and premotor areas (phonological route). A phoneme-linked visual image of kana or a syllable is accessed in the angular gyrus and the adjoining lateral occipital gyri and then the visual information of kana joins the orthographic route. Kanji characters that are graphically complex and have multiple stroke sequences depend more on the orthographic route, whereas kana characters that link directly to phonemes and have a graphically simple configuration depend less on the orthographic route. This hypothesis is consistent with recent neuroimaging findings (see Fig. 1 of Nakamura et al. [26]) that (i) kanji character writing and recall activated more extensive areas in the posterior inferior temporal cortex and superior parietal lobule than kana character writing and recall, and (ii) kanji writing and recall activated an extensive area of the posterior middle frontal gyrus (end of the orthographic pathway, Area 6), whereas kana writing and recall activated a limited area of the frontal operculum (end of the phonological pathway, Area 44/45). Recent neuropsychological findings from Western countries also support the view that the posterior inferior temporal cortex is a site for the orthographic lexicon for words [31] and additionally the posterior middle and inferior frontal gyri (Areas 44/45 and 6) and angular gyrus (Area 39) are concerned with accessing orthography [12,13].

A weak point of this hypothesis is that it does not take into account the features of apraxic agraphia, i.e. grapheme deformity and the above (3), following a superior parietal lobule lesion. Thus, it should be determined which lesion in the superior parietal lobule is responsible for grapheme deformity, disordered writing stroke sequences, and impaired recall of word images, respectively. Here, we report four patients with parietal writing impairment: isolated character deformity with or without limb apraxia, agraphia with character deformity and a stroke sequence disorder, and agraphia with a stoke sequence disorder and marked character deformity and imagery deficits (impaired character recall). We correlated the writing symptoms with the lesions and suggested anatomical substrates for these disturbances.

## 2. Materials and methods

### *2.1. Patient profiles*

Patient 1. In December 2001, a 36-year-old naturally left-handed man (laterality quotient by Edinburgh Handedness Inventory [28], -63), senior high school graduate and office worker trained to write with both hands in primary school, noted numbness of the left arm. He could not grasp anything. He was admitted to our hospital having been diagnosed with a cerebral infarction on CT. Neurological and neuropsychological examinations showed weakness of the left upper extremity, paresthesia with cheiro-oral topography that affected the left palm and oral corner, pseudoathetosis of the third to fifth fingers, tactile agnosia resulting from impaired combined sensations (texture recognition, two-point discrimination and graphesthesia) and limb apraxia of the left hand. His digit span forward score was 6, and backward 4. In two weeks his grip strength recovered, paresthesia was limited to the third to fifth fingers, and he could barely write, although limb apraxia still persisted.

The Western Aphasia Battery (WAB; Japanese edition) administered 13 days after onset showed no apha-

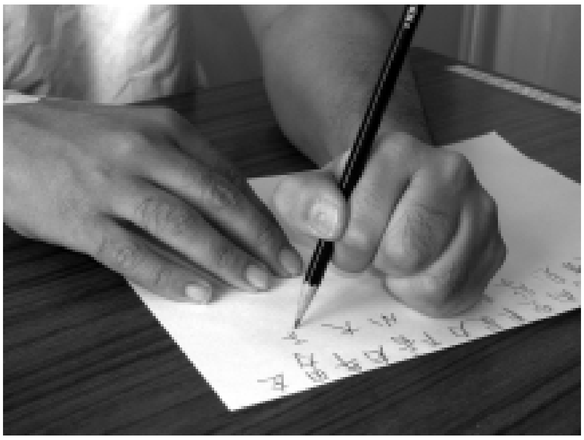

Fig. 1. Abnormal writing postures in patient 1 with “somesthetic dysgraphia.” Patient 1 wrote characters holding the pencil with the intact left thumb and index finger. He had tactile agnosia resulting from impaired combined sensations and limb apraxia in the left hand.

sia, but he wrote characters poorly (Table 1). He held the pencil in an awkward manner and wrote slowly and laboriously with the intact thumb and index finger (Fig. 1), which resulted in deformities of the written characters such as elongation and interruption of the lines. He formed better characters when copying a sentence than when writing in response to dictation. The patient could naturally hold the pencil and write kanji and kana words with the right hand without character distortion. Limb apraxia and tactile agnosia disappeared in a month and the left hemiparesis recovered, but paresthesia of the third to fifth fingers and impaired two-point discrimination still continued four months after onset.

MRI seven days after onset revealed high intensity areas in the right postcentral gyrus posterolateral to the precentral knob (cortical hand area above the operculum) [46], cortical and subcortical structures in the supramarginal and posterior middle temporal gyri (Fig. 2-1). There was another high intensity area in the right middle occipital gyrus that suggested an old infarction. Carotid angiography disclosed an occlusion of the horizontal portion (pars sphenoidalis) of the right middle cerebral artery.

Patient 2. In December 2001, a 74-year-old right-handed man, university graduate and retired office worker, noticed his right arm was weak and consulted our department. He was diagnosed as having a cerebral hemorrhage in the left parietal lobe on CT. He was admitted to the Department of Neurology at our hospital. On admission the patient showed slight weakness and paresthesia of the right arm, and tactile agnosia of the right hand resulting from deficient combined sensations (texture recognition, two-point discrimination and graphesthesia). He stated that everything he touched felt metallic and hard. There was no limb apraxia. He wrote kanji characters poorly, but recovered this ability considerably within ten days.

WAB ten days after onset showed no aphasia, but the patient wrote sentences slowly: he wrote only two sentences in three minutes when writing spontaneously, which resulted in a lower score for writing (Table 1). Some kanji and kana characters were poorly formed when writing spontaneously and writing in response to dictation, but much improved when copying a sentence. One month after onset, paresthesia was limited to the third to fifth fingers and pseudoathetosis was observed in the fourth finger. Writing impairment recovered to within the normal range three months after onset.

MRI eight days after onset revealed a high intensity on T1- and a low intensity on T2-weighted images in the localized area of the left postcentral gyrus posterior to the precentral knob (Fig. 2-2). There was mild enlargement of the left lateral ventricle.

Patient 3. In September 1996, a 58-year-old right-handed man, senior high school graduate and tie maker, found that he could not move his right arm, had lost his sense of touch with it, and felt as if it was another person’s. Ten minutes later when the motor weakness and sensory disturbance were recovered to some extent, he misspelled Japanese characters, even though he could recall their visual images. When he referred to a textbook, he could copy the characters correctly. Two days later, he could not knot a tie, and felt as if his right leg was covered with thin cotton. The patient was admitted to the Department of Neurology, University of Tokyo Hospital having been diagnosed with a cerebral infarction in the left parietal lobe on CT. Neurologi-

Table 1
Standard neuropsychological test scores

| Patient (age) | 1 (36) | 2 (74) | 3 (58) | 4 (58) |
|---|---|---|---|---|
| WAIS-R | | | | |
| Verbal IQ | 89 | 107 | 116 | 94 |
| Performance IQ | 73 | 94 | 108 | 65 |
| Picture Completion | 6 | 11 | 12 | 8 |
| Picture Arrangement | 5 | 6 | 12 | 6 |
| Block Design | 7 | 11 | 9 | 3 |
| Object Assembly | 9 | 8 | 11 | 3 |
| Digit Symbol (raw score) | 4 (35) | 9 (29) | 12 (50) | 4(15) |
| WAB | | | | |
| Spontaneous speech | | | | |
| Information content (/10) | 10 | 10 | 10 | 10 |
| Fluency (/10) | 10 | 10 | 10 | 10 |
| Naming total (/10) | 9.9 | 8.8 | 9.7 | 8.9 |
| Repetition (/10) | 10 | 10 | 9.8 | 10 |
| Comprehension total (/10) | 10 | 10 | 9.95 | 9.8 |
| Reading total (/10) | 9.5 | 10 | 9.2 | 8.8 |
| Recognition of orally spelled kanji (/6)# | 2 | 6 | 1* | 0* |
| Oral spelling of kanji characters (/6)# | 5 | 6 | 3 | 0* |
| Writing total (/10) | 10 | 8.6 | 8.6 | 3.15 |
| Copying (/10) | 10 | 10 | 10 | 10 |
| Kanji word writing from dictation (/6) | 6 | 6 | 4.5 | 1* |
| Kana word writing from dictation (/6) | 6 | 6 | 5.5 | 0* |
| Dictated kana characters (/5) | 5 | 5 | 5 | 0* |

*Subtest score is more than 2SD below the normal mean [42].
#Recognition of orally spelled kanji denotes that a patient is asked to guess a kanji character (e.g. 鳴 [naku], sing) from the two components (口 [kuchi], mouth, and 鳥 [tori], bird) spoken by the examiner. Oral spelling of kanji characters denotes that a patient is asked to describe two components (e.g. 女 [onna], woman, and 市 [ichi], city) of a kanji character (姉 [ane], sister) spoken by the examiner. These two tasks require mental imagery of a kanji character.

cal and neuropsychological examinations at this time showed no motor or sensory deficit, but impairments in short-term memory (digit span forward score was four), writing and mental arithmetic.

WAB administered four days after onset showed agraphia and an impairment of finger identification (Table 1). Writing errors included an impairment of kanji character recall, sometimes leading to neologisms, and literal paragraphia for kana. He used trial and error, particularly when writing kanji characters. Recognition of orally spelled kanji characters was significantly poor (for an example of this test, see the footnote of Table1), suggesting letter imagery deficits (impaired character recall). Copying of a sentence was perfect and orthographically improved compared to dictation, but he wrote stroke by stroke, referring to the sample. The patient complained that he could not write characters as he imagined. We did not examine his typing performance. There was no apraxia. Knotting a tie became possible as he practiced again. Calculation was slightly impaired in another calculation test between two digits and one digit [37] (mental arithmetic, 38/40; written calculation, 39/40). Impaired short-term memory and acalculia recovered to a normal level after three months, but the patient stated that he had difficulty writing characters over the next few years.

MRI one month after onset disclosed a high intensity area posterior to the left postcentral sulcus, including the superior parietal lobule and part of the supramarginal gyrus, that extended deep along the intraparietal sulcus to the left lateral ventricle on T2-weighted images (Figs 2–3). There were also small high intensity spots in the central semiovale on both sides and mild enlargement of the lateral ventricles that was more pronounced on the left.

Patient 4. In July 2001, a 58-year-old right-handed man, retired storekeeper, noticed convulsive movements in the right extremities and found difficulty writing. He could not make discrete finger movements or reach a glass on a table. He had sustained a cerebral hemorrhage in the left occipital lobe in January 1998 and developed right homonymous hemianopia and transient alexia. CT showed a low density area around the old infarction in the left occipito-parietal area. The patient was admitted to the Department of Neurology at our hospital having been diagnosed with a cerebral

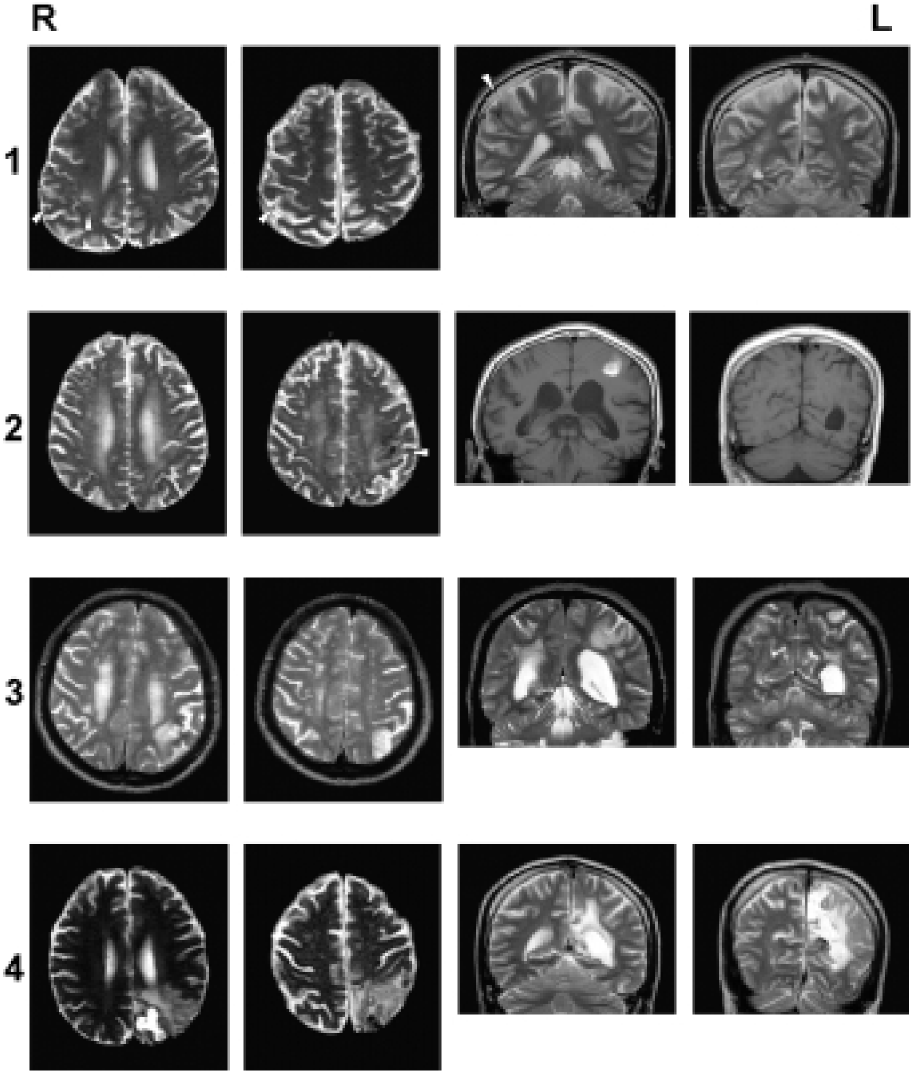

Fig. 2. MRI axial and coronal images of patients 1 to 4. Number corresponds to the patient number. Axial slices are at the level of upper part of the lateral ventricle and the intraparietal sulcus, and coronal slices area at the level of the posterior horn of the lateral ventricle and the angular gyrus. 2-1: High intensity areas were noted in the right postcentral gyrus posterolateral to the precentral knob, cortico-subcortical structures in the supramarginal gyrus on T2-weighted images (arrowhead, Time of repetition [TR] / Time of echo [TE] = 7999msec/104msec). These lesions were identified with diffusion-weighted images. On the coronal view, the postcentral gyrus lesion extended deep to the lateral ventricle (TR/TE = 4000/83). 2-2: A low intensity area suggestive of hemorrhage was localized in the left postcentral gyrus posterior to the precentral knob on T2-weighted images (arrowhead, TR/TE = 7999/101). On the T1-weighted planes, a high-intensity hemorrhage was noted over the posterior horn of the lateral ventricle (TR/TE = 500/14). 2-3: A high intensity area posterior to the postcentral sulcus extended deep along the intraparietal sulcus to the lateral ventricle on T2-weighted axial images (TR/TE = 3500/96). 2-4: A thin high intensity area was located in the occipital and parietal lobes that extended anteriorly to the postcentral sulcus, surrounding an old infarction (thick intensity area) in the medial occipital lobe on T2-weighted axial images (TR/TE = 7999/101). The new lesion involved the superior parietal lobule, angular gyrus and precuneus that extended posteriorly to the superior occipital gyrus on coronal images (TR/TE = 4000/84).

infarction. Neurological and neuropsychological examinations showed (1) right homonymous hemianopia, later confirmed by Goldmann perimetry, (2) Barré's hand pronation sign of the right upper extremity and the right extensor plantar response, (3) agraphia for kanji and kana caused by impaired character recall, (4) an impairment of short-term memory (digit span forward score was four), (5) acalculia, and (6) a visually guided reaching disturbance of the right hand for objects in the left upper quadrant visual field.

WAB administered seven days after onset showed severe agraphia, characterized by impaired character recall of both kanji and kana, poor grapheme formation and abnormal stroke sequences (Table 1). The patient wrote characters slowly and laboriously. He could not recognize orally spelled kanji characters or spell aloud auditorily presented kanji characters, which resulted in a low score for reading. He showed literal paragraphia and impaired recall even for dictated single-kana characters. However, the patient could type kana words that he could not write in response to dictation. He copied sentences perfectly, but he wrote stroke by stroke, referring to the sample. Most written characters were poorly shaped, like geometrical figures, as if they had been written by a child. Copying of line drawings (e.g. a cube) was also good, but spontaneous drawing without a sample was done out of perspective, suggesting he also had impaired mental imagery for non-linguistic figures. The visually guided reaching disturbance disappeared 15 days after onset but agraphia persisted even one year post-onset.

MRI three days after onset revealed a high intensity area with a marginal low intensity in the left cuneus, lingual gyrus and forceps major in the occipital lobe, suggesting an old hemorrhagic infarction, and surrounding it a thin high intensity in the left occipital and parietal lobes, including the angular gyrus, precuneus and superior parietal lobule that extended forward to the postcentral sulcus on T2-weighted images (Figs 2–4).

### 2.2. *Neuropsychological tests*

To evaluate basic cognitive functions and language abilities, we administered WAIS-R and WAB to each patient (Table 1). Subtest scores of performance IQ in WAIS-R are shown to reveal cognitive and motor skills. To evaluate each patient's reading and writing ability quantitatively, we gave them a special test (Table 2). The task was to read aloud 100 single-character kanji and the kana transcription of kanji characters, and to write the same 100 dictated kanji and kana [36], all of which are taught in the first three years of primary school in Japan. The performances of patients 1 and 4 were recorded with a digital video camera and writing stroke sequences were analyzed. Patient 4 was asked to copy the test characters further.

Correct answers and time for reading and writing were counted. In the writing test, errors were subdivided into non-responses, partial responses (incomplete characters or words), constructional errors (omission or addition of a component of a character), visual errors (substitution of another visually similar character), phonological errors (substitution of another character with a different phonetic value), semantic errors (substitution of another word semantically associated with the correct word), etc. according to our previous classification [38] (Table 3). Trial and error and abnormal stroke sequences were evaluated for all characters tested. Deformity was defined as the malformation of graphemes (characters), and was classified into disproportion (imbalanced size of each component in a character or word), dislocation (wrong position or direction of each component or stroke), line distortion (twitching of a straight line), curve distortion (twitching of a curve), elongation of a line or stroke, and interruption of a line or stroke. Deformity was evaluated only for correct responses. If a character had two or more deformity types, each type was counted separately.

Whether a character is regarded as being well formed or not depends on an examiner. Thus, we first chose and classified deformed characters primarily on the basis of the patients' evaluation. In order to evaluate deformity more objectively, three authors (Y.O., G.N. and T.M.), all native Japanese, assessed the patients' writing independently, and a character was counted only if two or more authors had the same evaluation of the deformity or deformity type. Deformed characters were chosen if two or more authors' assessment was identical irrespective of the assessed deformity type. If the order of writing stroke sequences was unusual and it was not caused by the patient's writing habit, the sequences were considered to be disordered. Impaired character recall (letter imagery deficit) was counted when the patient did not make any response in writing to dictation (non-response in Table 3). We adopted the following criteria to diagnose apraxic agraphia: (i) production of illegible graphemes in writing that cannot be accounted for by sensorimotor dysfunction [1,30,32], (ii) grapheme production improves with copying [32], (iii) preserved oral spelling or typing [32], and (iv) disordered writing stroke sequences [29].

## 3. Results

Deformity analyses below are mainly based on the authors' objective assessment, if not described in particular. Patient 1 took slightly longer to write kanji and kana, although the scores were nearly perfect. Deformity involved dislocation, elongation and interruption, and was more frequently observed in kanji (Fig. 3). The patient took the same test four months after onset, when he still had paresthesia limited to the third to fifth fingers and a minimal reduction in grip strength. Re-

Table 2
Reading and writing performances of 100 single-character kanji and the kana transcription

| Patient | 1 | 2 | 3 | 4 | Controls ($n = 11$) | |
|---|---|---|---|---|---|---|
| | | | | | Mean score (SD) | Mean time (SD) |
| Kanji reading | 100 (48 s) | 100 (1 min 26 s) | 100# | 100 (4 min 8 s*) | 99.6 (1.2) | 1 min 32 s (32 s) |
| Kana reading | 100 (1 min 28 s) | 100 (1 min 13 s) | 100# | 100 (2 min 27 s*) | 99.6 (0.5) | 1 min 13 s (24 s) |
| Kanji writing | 99 (17 min 15 s*) | 97 (23 min 2 s*) | 95 (19 min*) | 28* (37 min 12 s*) | 95.9 (3.0) | 10 min 11 s (2 min 16 s) |
| Kana writing | 100 (12 min 10 s*) | 100 (20 min 28 s*) | 100 (16 min*) | 50* (63 min 8 s*) | 99.3 (0.9) | 8 min 7 s (1 min 47 s) |

Normal controls [38] were 10 men and one woman, ages 61 to 78, mean 68 years old, senior high school or university graduate volunteers who had no past history of neurological disorders. Note that writing speed was not exactly reflected in the time spent because word-finding pauses were also included in the time for patients 3 and 4. In patient 4, however, writing speed was obviously slow.
#In patient 3, data for reading time were missing.
*More than 2SD above (for time) or below (for score) the normal mean.

sults showed general improvement, but he was aware that eight kanji characters and seven kana characters were poorly written. Patient 2 took more than twice as long to complete the tasks as the control subjects. Deformity consisted mainly of dislocation and elongation in kanji, and curve distortion in kana, although the patient was more aware of disproportion and line distortion in kanji and dislocation in kana. The patient complained that he could not write characters as he imagined, showing imbalanced kanji characters and elongation. Although paresthesia in the third to fifth fingers persisted, a re-examination three months after onset showed almost perfect recovery in writing. Patient 3 spent more time writing both kanji and kana because he wrote the wrong stroke sequences by trial and error: He stopped writing in the middle of a kanji character and wrote it again from the beginning, even though the character was written correctly in six trials. He also added a line to an incomplete kanji character after writing it in two trials. According to the patient's evaluation, malformation of graphemes was more pronounced in kana writing that required for curve drawing [20]. Patient 4's performance was the lowest and he needed the longest time of the four patients. Kanji writing was more impaired than kana writing. Most errors were non- and partial responses resulting from impaired recall of both kanji and kana characters, and katakana (a form of kana that is used primarily for representing loan words) substitution in kana. To determine the effects of visual complexity, concreteness, familiarity (how often a person has seen or used a word) and frequency of writing single-character kanji, we divided test characters into two groups (above or under a median) nearly equal in number: a more complex (more writing stroke sequences), concrete, familiar or frequent group and a less complex, concrete, familiar or frequent group according to our previous study [39]. Correct scores for two groups each were significantly different in complexity ($p = 0.007$ by Fisher's exact method) and familiarity ($p = 0.026$ by Fisher's exact method), i.e. less complex and more familiar characters were written more easily. The patient uttered a word repeatedly when he had difficulty writing the word (phonological facilitation [35]). There were a few traces of trial and error written on a piece of paper in kanji, but in fact the patient did many rehearsals, moving the pencil over the paper before writing down a character. Disordered stroke sequences were more evident in kanji writing, when the patient appeared to forget the stroke sequences of some kanji. Deformities included dislocation in both kanji and kana and curve distortion in kana. Copying of the 100 kanji characters improved overall grapheme formation (He thought that nine of 11 deformed characters in writing in response to dictation were written better when copying), but there were still abnormal graphemes. He also wrote strokes as if he had drawn geometrical figures (abnormal stroke sequences in copying kanji: 22/100). A re-examination eight months after onset showed considerable recovery (score and time: kanji 38/100 and 38 min, kana 69/100 and 44 min), but the patient still wrote poorly formed characters with disordered stroke sequences.

Table 4 summarizes the writing features and other clinical profiles for our four patients and reported patients with Japanese parietal agraphia [14,18,21,23,29, 45]. It is noteworthy that performance IQ was equal to or less than verbal IQ in WAIS-R for all patients. In patients 3 and 4 with a superior parietal lobule insult, Block Design and Object Assembly were performed particularly poorly. Thus, lower performance IQ probably reflects visuospatial cognitive dysfunction of the "left" superior parietal lobule. Figure 4 shows the individual lesions of patients in Table 4 with abnormal character shape formation (our patients 1–4) [14,21,23, 29] disordered writing stroke sequences (our patients 3,4) [14,18,23,29] and character imagery deficits (our patients 3,4) [14,18,21,23,45] mapped onto an axial plane through the intraparietal sulcus [8]. Lesion sites

Table 3
Types of writing error in 100 single-character kanji and the kana transcription

| Patient | 1 | 2 | 3 | 4 |
|---|---|---|---|---|
| Kanji total errors | 1 | 3 | 5 | 72 |
| Non-response | 0 | 2 | 1 | 51 |
| Partial response* | 0 | 0 | 1 | 9 |
| Constructional* | 0 | 0 | 0 | 8[a] |
| Neologism | 0 | 1 | 2 | 2 |
| Visual* | 1 | 0 | 1 | 0 |
| Unrelated* | 0 | 0 | 0 | 2 |
| Trial and error# | 0 | 1 | 15 | 1 |
| Abnormal stroke sequences# | 0 | 0 | 4 | 9 |
| Deformity total# | 15 (28) | 33 (16) | 3 (5) | 11 (5) |
| Disproportion | 1 (0) | 5 (0) | 0 (0) | 0 (0) |
| Dislocation | 5 (10) | 6 (4) | 2 (1) | 10 (3) |
| Line distortion | 2 (1) | 11 (1) | 1 (1) | 0 (0) |
| Curve distortion | 0 (0) | 3 (0) | 0 (0) | 1 (0) |
| Elongation | 4 (7) | 8 (3) | 0 (1) | 0 (0) |
| Interruption | 3 (1) | 0 (0) | 0 (0) | 0 (0) |
| Kana total errors | 0 | 0 | 0 | 50 |
| Non-response | 0 | 0 | 0 | 8 |
| Partial response** | 0 | 0 | 0 | 21 |
| Constructional** | 0 | 0 | 0 | 6[b] |
| Phonological** | 0 | 0 | 0 | 3 |
| Phonological and visual** | 0 | 0 | 0 | 2 |
| Katakana substitution** | 0 | 0 | 0 | 10 |
| Trial and error# | 0 | 0 | 15 | 10 |
| Abnormal stroke sequences# | 0 | 0 | 2 | 1 |
| Deformity total# | 4 (22) | 17 (12) | 12 (5) | 6 (6) |
| Disproportion | 0 (1) | 0 (0) | 0 (0) | 0 (0) |
| Dislocation | 2 (4) | 9 (0) | 2 (1) | 2 (2) |
| Line distortion | 0 (0) | 1 (2) | 1 (0) | 0 (0) |
| Curve distortion | 2 (6) | 5 (6) | 8 (2) | 4 (2) |
| Elongation | 0 (3) | 2 (1) | 0 (0) | 0 (0) |
| Interruption | 0 (1) | 0 (0) | 1 (0) | 0 (0) |

Data were based on the results of the kanji and kana writing test (Table 2).
*Partial response: a component of a kanji character is correct. Constructional response: omission or addition of a component of a kanji character. Visual errors: substitution of another visually similar character, e.g.緑 ([midori], green) → 縁 ([en], margin). Unrelated response: substitution of another kanji that has no visual or phonological similarity to the correct answer, e.g夏 ([natsu], summer) → 勧 ([susumeru], recommend).
**Partial response: one character or more in a kana word is correct. Constructional response: omission or addition of a component of a kana. Phonological response: one or more characters of a kana word were substituted for other kana (phonemic paragraphia), e.gしる ([shiru], know) → ひる ([hiru], noon). Phonological and visual response: changing one character into another visually similar to the target character, e.g. くろい ([kuroi], black) → くるい ([kurui]). Katakana substitution: substitution of Katakana (a form of kana that is used primarily for representing loan words) that has the same phonemic value, e.g.くるま ([kuruma], wheel) → くルま ([kuruma]).
[a]Constructional errors consisted of five deletions of one stroke and three interruptions of one stroke.
[b]Constructional errors consisted of five deletions and one addition.
#Trial and error and abnormal stroke sequences were evaluated for all characters tested. Trial and error denotes an attempt to write an incorrect or incomplete character repeatedly. Deformity was evaluated only for correct responses. Scores denote the patient's subjective evaluation and three authors' assessment (in the parentheses). In the three authors' assessment, a character was counted only if two or more authors had the same evaluation of the deformity or deformity type. Deformed characters were chosen by two or more authors' evaluation whether or not the deformity type assessment was identical among authors. Eventually, the number of deformity total exceeded the sum of each deformity type in all patients. If a character had two different deformity types, these two were counted separately. Disproportion refers to the imbalanced size of each component in a character or word. Dislocation is the wrong position or direction of each stroke. Distortion is the twitching of a straight line or curve. Elongation and interruption are of a line or stroke.

were delineated with a sheet of lettering screen and each lesion image cutout was overlapped on the standard axial plane at the level of the intraparietal sulcus. All three symptoms involved lesions surrounding the intraparietal sulcus (overlapped area). In particular, the overlapping appears more pronounced in the anterior half of the intraparietal area in disordered stroke sequences (middle), and extensive areas including the supramarginal gyrus or the precuneus in character imagery deficits (right side).

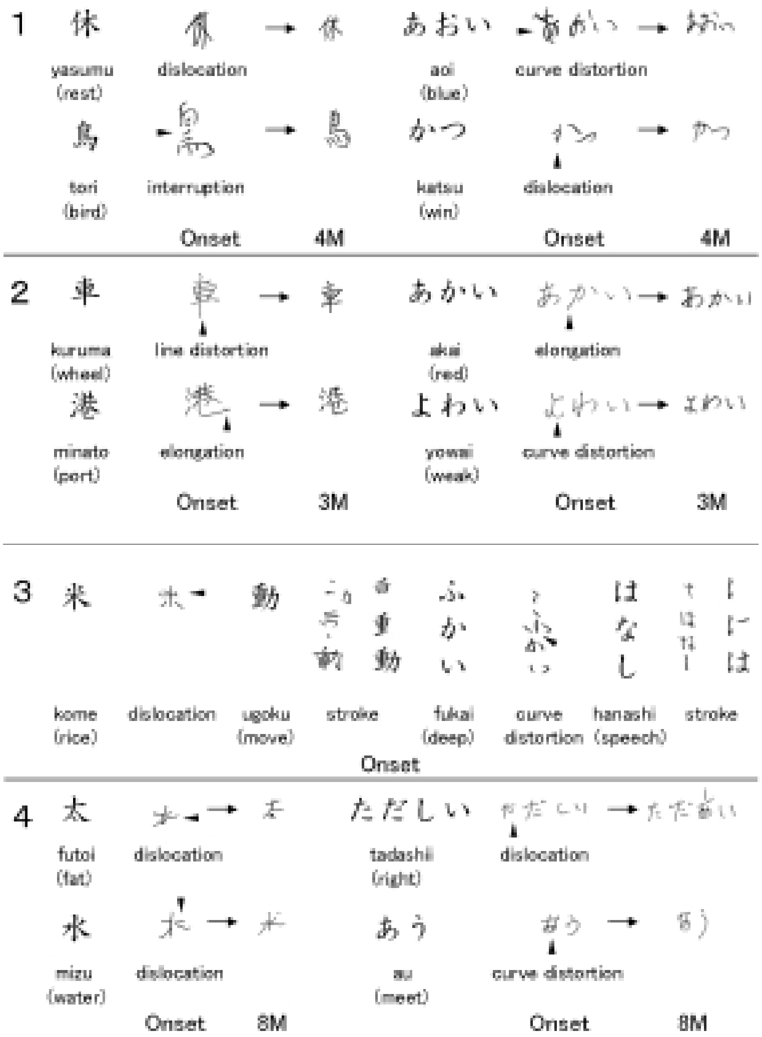

Fig. 3. Examples of abnormal grapheme formation and disordered writing stroke sequences in patients 1 to 4. Number corresponds to the patient number. An arrow head denotes the part of deformity. All samples of the deformity type were based on three authors' objective assessment. In patients 3 and 4, stroke sequence errors were frequently accompanied by trial and error, as in patient 3 (orthodox stroke sequences are shown on the right). Patients 1, 2 and 4 underwent the same test in a follow-up study. Abbreviations. 3M: three months after onset, stroke: stroke sequence disorder.

## 4. Neuroimaging study

All four patients underwent SPECT with the $^{99m}$Tc-ethylcysteinate dimer (ECD-SPECT) 17 days (patient 1), 21 days (patient 2), 33 days (patient 3) and 21 days (patient 4) after onset. SPECT data were transformed into the Analyze Format and were normalized, smoothed and corrected for inter-laboratory differences with a 3-dimensional conversion map [24]. For this system, realignment, spatial normalization and smoothing are essentially the same as those of Statistical Parametric Mapping (SPM) Version 1999, and the statistical significance was determined with SPM 96 for Windows. The data were compared with those of the normal subject database of the same generation at the National Center of Neurology and Psychiatry, Tokyo ($n = 20$). Areas showing a significant decrease in cerebral blood flow (corrected $p < 0.01$) were rendered on standard brain surface images (Fig. 5). Reduced blood flow was found in the postcentral gyrus in all four patients. In addition, the precentral gyrus was affected in patient 1 with the left hand weakness and limb apraxia. The superior parietal lobule was involved in patients 1, 3 and 4, the angular gyrus in all patients, and the supramarginal gyrus in patients 1 and 3.

Table 4
Summary of the reported cases of Japanese parietal agraphia and the present patients

| Authors | | Writing | | | | | Apraxia | Other symptoms | WAIS-R | Lesion |
|---|---|---|---|---|---|---|---|---|---|---|
| | | Shape | Speed | Stroke | Imagery | Copying | | | | |
| Kawamura [18] | Pt 1 | N | ? | I | I | N | – | LR, FA, AC | V 97, P 97 | SPLa, SMG |
| | Pt 2 | N | ? | I | I | N | IMA | LR, FA, AC | V 88, P 74 | SPLa, SMG |
| Kojima et al. [21] | Pt 1 | I | ? | N | I | I | IA | CD, USN, TD | V 98, P 68 | SPLp, Cu, Sup O |
| Ishiai et al. [14] | Pt 2 | I | I | I | I | I | – | – | V 107 | SPLap |
| Toyokura et al. [45] | | N | ? | N | I | N | – | – | not done | sSPLap, sAG, Cu |
| Maeshima et al. [23] | | I | I | I | I | I | – | – | V 94, P68 | SPLap, AG |
| Otsuki et al. [29] | | I | ? | I | N | I | – | – | V 109, P 100 | SPLa |
| Our Pt 1 | | I | I | N | N | sl. I | LA | TA | V 89, P 73 | Post C, SMG |
| Pt 2 | | I | I | N | N | sl. I | – | TA | V 107, P 94 | Post C |
| Pt 3 | | sl. I | N | I | sl. I | N | – | FA, AC | V 116, P108 | SPLap, SMG |
| Pt 4 | | I | I | I | I | I | – | AC, RD | V 94, P 65 | SPLap, SMG, AG, Cu, Sup O |

The evaluation of shape (grapheme formation), speed (writing speed), stroke (writing stroke sequences) and imagery (non-response that is due to poor character recall) in writing in response to dictation, and copying (copying the visually-presented characters) were based on the authors' assessment described in the original papers.
Abbreviations. I, impaired; N, normal; sl., slightly; V, verbal IQ; P, performance IQ; Pt, patient; LR, left-right disorientation; FA, finger agnosia; IA, ideational apraxia; IMA, ideomotor apraxia; LA, limb apraxia; CD, constructional disorder; USN, unilateral spatial neglect; TD, topographical disorientation; AC, acalculia; RD, visually guided reaching disturbance; SPL, superior parietal lobule; a, lesion extending anteriorly to the postcentral sulcus; p, lesion extending posteriorly to the parieto-occipital junction; ap, lesion extending both anteriorly and posteriorly; s, sub-cortical; AG, angular gyrus; Cu, cuneus and precuneus; Sup O, superior occipital gyrus; SMG, supramarginal gyrus; Post C, postcentral gyrus.

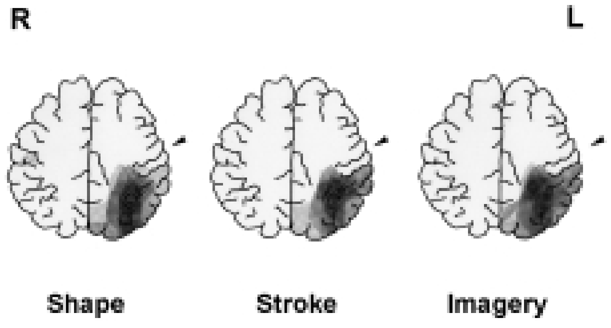

Fig. 4. Overlapped lesions in the parietal lobe in patients with abnormal character shape formation, disordered writing stroke sequences and character imagery deficits (impaired character recall). An arrow head denotes the central sulcus. The axial image through the intraparietal sulcus was drawn from Damasio [8]. All three symptoms involved lesions surrounding the intraparietal sulcus. The overlapping was more pronounced in the anterior half of the intraparietal area in disordered stroke sequences (middle), and extensive areas in the parietal lobe in character imagery deficits (right side).

## 5. Discussion

The four patients reported here all exhibited writing impairments from parietal lobe lesions. Patient 1 was left-handed. As his impairment, however, was solely caused by damage to the sensorimotor area, the discussion could be applied to right-handers. In addition, a slightly shortened digit span for his age (this patient had a small infarction in the supramarginal gyrus) suggests that patient 1's language function was performed in the right hemisphere. Patient 3 had a bilateral lacunar infarction in the centrum semiovale, but this old infarction had little effect on the patient's general cognitive function, as revealed by WAIS-R. Patient 4 had sustained a cerebral hemorrhage in the left medial occipital gyri and the right homonymous hemianopia had remained. Additional infarction in the adjoining area in the occipital and parietal lobes produced severe agraphia with impaired character recall. Thus, this new lesion in the superior parietal and parieto-occipital areas was critical in the occurrence of agraphia, although his reading and writing speed were also affected by hemianopia (discussed in 5.4).

Since apraxic agraphia presupposes normal sensorimotor function [1,32], it is clear that patients 1 and 2 did not have apraxic agraphia. Instead, they demonstrat-

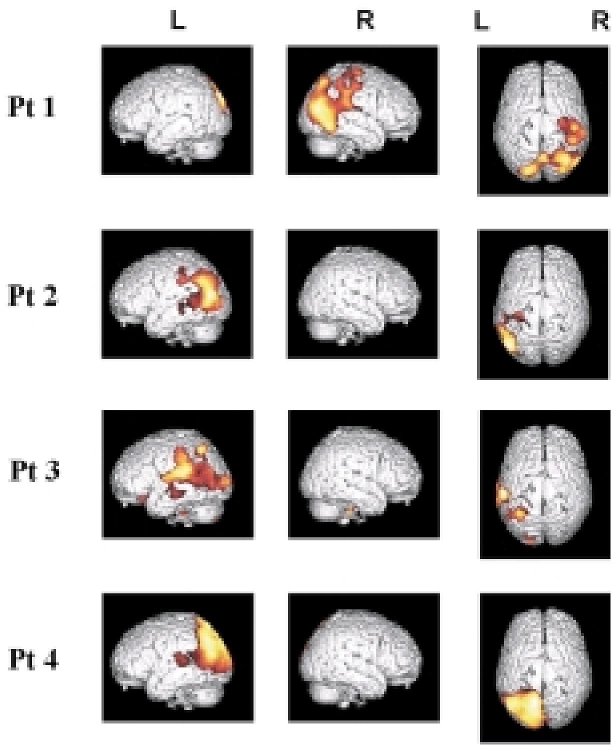

Fig. 5. Decreased blood flow in the four patients determined with SPM 96 in ECD-SPECT. Patient data were compared with those of a normal database of the same generation in the National Center of Neurology and Psychiatry, Tokyo ($n = 20$). A significant decrease in cerebral blood flow was shown (corrected $p < 0.01$). Reduced blood flow was found in the postcentral gyrus in all four patients. The superior parietal lobule was involved in patients 1, 3 and 4. Note that the localized region of the somesthetic sensory area was affected in patients 1 and 2, who exhibited grapheme deformity.

ed that dysgraphia, i.e. abnormal grapheme formation, occurs because of a postcentral gyrus lesion, regardless of accompanying limb apraxia.

Patients 3 and 4 had apraxic agraphia in the sense that they showed (i) abnormal grapheme formation, (ii) improvement in poorly formed characters when copying, (iii) preserved kana word typing (patient 4), and (iv) disordered writing stroke sequences. Impaired character/letter recall (letter imagery deficit) is sometimes observed in apraxic agraphia [7,22]. Even if the patient does not spell aloud completely a word that he cannot write, this does not exclude the diagnosis of apraxic agraphia. Recognition of orally spelled kanji characters and oral spelling of kanji characters were poor in patient 4 (Table 1), suggesting kanji character imagery deficits. Recognition of orally spelled kanji characters was also poor in patient 3. In addition, the fact that he wrote a character again from the beginning, even though the character was written correctly, suggests that the visual image of the character was unstable. In short, patient 3 had slightly, and patient 4 severely, impaired mental imagery of kanji. We discuss the anatomical substrates of mental imagery deficits in 5.3.

According to a cognitive neuropsychological model [10,30], the writing process can be subdivided into central (or linguistic) and peripheral (or motor) components. Central components are responsible for selecting the appropriate words for written output, whereas peripheral components are responsible for converting orthographic information into handwriting movements. We prefer the terminology of linguistic vs. motor to that of central vs. peripheral because all these processes occur in the "central" nervous system. From this point of view, all four patients had motor impairments of handwriting, and in addition, patients 3 and 4 had linguistic disorders of word/character recall.

### 5.1. Abnormal grapheme formation

Patients 1 and 2 showed that character shape was disturbed with a postcentral gyrus lesion, with or without limb apraxia. Abnormal grapheme formation due to a postcentral gyrus lesion has not been reported previously. It is clear that the deficit lay in the motor aspect of handwriting. However, these cases are not delineated in the cognitive neuropsychological model of spelling and writing [10,30,32]. It is likely that sensory and kinesthetic feedback from the writing hand ascended to the opposite somesthetic sensory or somatosensory hand area (Areas 1,2 and 3), but the partial damage had an inappropriate effect on the motor programming [32] (or graphic innervatory patterns [30], anatomically Area 4 hand area) or directly influenced the corticospinal tract. It is known that descending fibers from the postcentral gyrus (Areas 1, 2, and 3) join the corticospinal tract from the precentral gyrus (Area 4) [34]. Functional neuroimaging studies also suggested the involvement of the postcentral gyrus in handwriting [4,16]. Thus, we designate this type of writing impairment "somesthetic dysgraphia."

It is difficult to differentiate the character deformity in patients 1 and 2 from that in patients 3 and 4 with apraxic agraphia. Analysis of writing errors, however, showed that in kanji writing dislocation accounted for many errors in patients 3 and 4, whereas there were many elongation errors other than dislocation in patients 1 and 2 (Table 3). This difference is probably because acquired information about spatial alignment of each component or stroke was disturbed in patients 3 and 4 (described in 5.6), whereas the deficit was limited to fine control of handwriting in patients 1 and 2.

Regarding the neural substrate, our patient 3 and reported cases [14,21,23,29] suggest that abnormal grapheme formation arises from lesions surrounding the intraparietal sulcus (Fig. 4, left side). In addition, graphemic distortion seems to be more pronounced when the parietal lobe lesion extends to the superior occipital gyrus and precuneus, as in our patient 4.

### *5.2. Writing stroke sequence disorder*

Disordered writing stroke sequences are another symptom that characterizes apraxic agraphia. This symptom is easily observed, especially in writing of kanji that have multiple stroke sequences [29], but it has also been reported in Western countries [6], where a video camera was used. Reported patients [14,18,23, 29] and our patients 3 and 4 suggest that a lesion in the intraparietal area, particularly the anterior part that extends forward as far as the postcentral sulcus, causes abnormal writing stroke sequences (Fig. 4, middle). Thus, an area adjoining the postcentral gyrus in the intraparietal sulcus seems to be critical to the occurrence of disordered writing stroke sequences.

### *5.3. Mental imagery deficit of kanji and kana*

We can see in Fig. 4 (right side) that letter/character imagery deficits (impaired character recall) were also attributed to the damage surrounding the intraparietal sulcus, but in some cases the lesion extended inferiorly to the supramarginal and angular gyri [18,23,45] or posteriorly to the superior occipital gyrus and precuneus [21,45]. The involvement of the inferior parietal and posterior occipital gyri was also observed in reports from Western countries [7,22], in which mental imagery of letters was impaired. Comparison of the symptom-to-lesion between patients 3 and 4 suggests that mental imagery is poorer as the lesion involves the angular, superior occipital and precuneus gyri.

In patient 4, impaired character recall was more prominent in kanji, but kana character recall was also disturbed, which was not observed in patients 1, 2 and 3 (Tables 1 and 2). Reported cases showed agraphia more impaired for kanji [14,17,23] and agraphia for both kanji and kana [17,21,29,45] with a superior parietal lobule lesion. This difference probably depends on whether the lesion extends inferiorly to the supramarginal gyrus or angular gyrus, or posteriorly to the parieto-occipital junction. A lesion in the posterior area of the superior parietal lobule alone or around the intraparietal sulcus can yield agraphia more impaired for kanji [14,17]. Furthermore, it is often seen that agraphia that impairs kana writing more than kanji writing occurs because of a supramarginal gyrus lesion [37,43], whereas agraphia that impairs kanji writing more than kana writing occurs because of an angular gyrus lesion [14,15,17,25, 38]. Since the inferior supramarginal gyrus is thought to be the phonological short-term-memory store [37] and kana characters directly represent a sequence of phonemes (consonant-vowel syllables), it is quite understandable that agraphia or literal paragraphia for kana occurs due to damage to the supramarginal gyrus. In this case, however, it should be noted that most of the errors were not impaired recall, but literal paragraphia [35,43]. Patient 1 with the supramarginal gyrus damage did not exhibit a phonological short-term memory impairment or kana agraphia. This is probably because the lesion was too small to produce symptoms (strictly speaking, his digit span forward 6 was slightly low for his age, as described). Hypoperfusion of the parietal lobe in the patient's SPECT image probably reflected relative ischemia caused by occlusion of the right middle cerebral artery.

Conversely, it is not clear why kanji writing is more disturbed than kana writing in an angular gyrus lesion.

We believe that orthographic information from the posterior inferior temporal cortex is sent by way of the angular gyrus (probably through the subcortical association fibers) to the superior parietal lobule, and this route is interrupted by damage to the angular gyrus, which produces kanji orthography recall deficits [35] (Fig. 6). Kana writing is also disturbed in an angular gyrus lesion, although there are few errors [14,15,17,25,38]. In our study [38], errors included phonological and constructional (omission or addition of a component of a kana character) errors.

The lesion extension to the parieto-occipital junction, i.e. the superior occipital and precuneus gyri, is probably another factor that influences the kanji vs. kana difference. As patient 4's kana writing errors consisted mostly of non- and partial responses (impaired recall) with a few phonological errors (literal paragraphia), it is clear that they were not attributable to dysfunction of the supramarginal gyrus or angular gyrus. Instead, the superior parietal lobule and the adjoining superior occipital and precuneus gyri seem to be concerned in recalling kana, as well as kanji, orthography. Damage to this area may give rise to imagery deficits of both kanji and kana (graphemic area in a broad sense, discussed in 5.6), but kanji are more affected because of their complex configuration. In support of this view, a visual complexity effect (less complex characters are recalled and written better than more complex characters) was observed in patient 4's performance in the 100 kanji writing test.

In summary, mental imagery may be more seriously affected as the lesion extends from the intraparietal area to the angular, superior occipital and precuneus gyri. Furthermore, the involvement of the supramarginal gyrus, angular gyrus or superior occipital and precuneus gyri can determine the severity of kanji or kana recall impairment. A lesion restricted to the anterior area of the superior parietal lobule may not cause a character recall impairment [29].

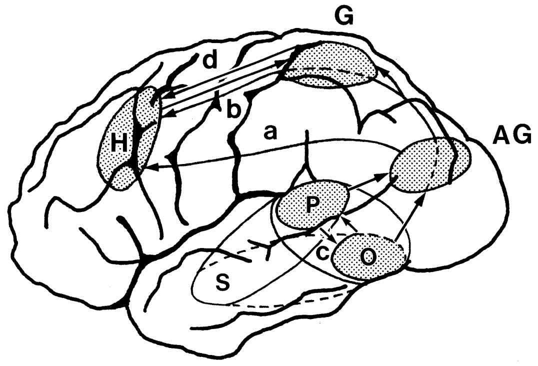

Fig. 6. Diagram of an anatomically based dual-route hypothesis for writing in response to dictation (modified from Sakurai et al. [35]). a. phonological pathway, b. orthographic pathway, c. interaction between phonology and orthography, d. interaction between parietal graphemic area and frontal hand area. Phonological information of a word goes from the primary auditory cortex (Heschl's gyri) and the posterior superior temporal gyrus (Wernicke's area) to the angular and supramarginal gyri and joins the arcuate fasciculus to travel to the frontal motor and premotor areas (a, phonological route). A phoneme-linked visual image of kana or a syllable is accessed in the angular gyrus and the adjoining lateral occipital gyri and then the visual information of kana joins the orthographic route to travel to the frontal cortex. The posterior superior temporal gyrus (P), where the phonological information of words is stored, and the posterior inferior temporal cortex (O; Brodmann Area 37), where the orthographic information of words is stored, have a reciprocal connection (c). Lexico-semantic information (S) is stored in extensive areas in the left temporal lobe, and can be accessed either through the posterior superior temporal gyrus during listening or through the posterior inferior temporal cortex during reading (illustrated in dotted lines). Orthographic information (visual images of kanji words and characters and also kana words) goes from the posterior inferior temporal cortex and proceeds upward under the angular gyrus and the superior parietal lobule to travel to the premotor hand area (H; Areas 44/45 and 6). This orthographic route (b) goes directly or indirectly to the hand area via the parietal graphemic area (G) where visuokinesthetic and sequential motor engrams for letters and words are stored. The graphemic area in a broad sense includes the inferior parietal lobule and parieto-occipital junction (superior occipital and precuneus gyri), in addition to the superior parietal lobule and intraparietal area, and stores visuospatial attributes of characters. Kanji characters that are graphically complex and have multiple stroke sequences depend more on the orthographic route, whereas kana characters that link directly to phonemes and have a graphically simple configuration depend less on the orthographic route. The parietal graphemic area and the frontal hand area (H) have a reciprocal connection (d).

### *5.4. Reduced writing speed*

Writing speed was obviously low for patient 1. Reduced writing speed was probably caused by weakness of the damaged hand or insufficient sensory and kinesthetic feedback from the damaged hand during writing because the WAIS-R Digit Symbol subtest performance was also poor in patient 1 (Table 1). Although patient 2 got a normal score compared with normal controls in the Digit Symbol subtest, the fact that his time for writing in the 100-kana writing test (mental imagery is less involved in this test) recovered three months later (from 20 to 14 min) suggests that patient 2 also had a low writing speed at disease onset solely because of insufficient sensory and kinesthetic feedback. Conversely, Patient 4 was extraordinarily slow at writing. One reason is that he had right hemianopia. Another reason is probably associated with the poor grapheme formation in apraxic agraphia. That is, because of a defective visuokinesthetic engram (sequential motor patterns) for words and letters, or more generally symbols, the patient could not write or spell words properly and smoothly, and this required a lot of time. Patients with apraxic agraphia who had a low writing speed as well as poor grapheme formation have been reported [14, 23].

### 5.5. Neuroimaging data

The SPECT data showed extensive areas of hypoperfusion in the temporo-parietal and parieto-occipital junctions in all patients (Fig. 5). For patient 1, occlusion of the right middle cerebral artery was associated with the hypoperfusion of these areas. For patients 2 and 3, left-side dominant enlargement of the lateral ventricles seemed to influence the blood flow of the temporo-parietal junction. As patients 1 and 2 did not show other cognitive impairment except for motor dysgraphia, hypoperfusion of the temporo-parietal and parieto-occipital areas is irrelevant to the symptoms. Blood flow reduction in the localized area of the sensorimotor cortex in patient 1 and the somesthetic sensory area in patient 2 supports our view that these areas are also involved in grapheme formation. Patient 3 had minimal impairment of character recall (letter imagery deficit). It is possible that decreased blood flow to the supramarginal and angular gyri had some effect on performance. Even so, however, this does not alter the conclusion that more extensive areas in the parietal and occipital lobes are needed to cause severe deficits of grapheme formation and mental imagery, which is clear from the comparison of blood flow images between patients 3 and 4.

Functional imaging studies have revealed activation of the posterior inferior temporal cortex, intraparietal area, superior parietal lobule, sensorimotor cortex, posterior middle frontal gyrus, dorsolateral prefrontal cortex, and supplementary motor area in a variety of writing tasks [4,16,26,27,44]. These activated areas include all the lesion sites of our patients. Among them, the posterior inferior temporal cortex and intraparietal area were also activated in mental recall of kanji and kana characters [26,27], suggesting that they were engaged in retrieval of orthography. Besides, the superior parietal lobule, sensorimotor cortex, posterior middle frontal gyrus and supplementary motor area were activated while writing in contrast to naming [4], suggesting that they were concerned with the motor aspect of handwriting. It should be noted that the superior parietal lobule or intraparietal area was activated during both word recall and handwriting, which implies that the intraparietal area plays an important role in word recall, orthography-to-motor transcoding and execution of handwriting movements.

### 5.6. Modified dual-route hypothesis for writing

To account for abnormal letter formation and letter imagery deficits (impaired letter recall) in apraxic agraphia, a graphemic area responsible for transcoding features of letters into graphemic production patterns [33] was considered to regulate both motor transcoding of letter features and letter imagery [7]. Apraxic agraphia was assumed to result from a disruption of the parietal graphemic area or disconnection of output from the graphemic area [32]. Another theory attributes the function of motor transcoding to the graphic motor programs that specify the sequence, direction and relative size of strokes [9,30]. It remains unclear whether grapheme formation and mental imagery are performed in an anatomically identical area. Our patients 3 and 4 suggest that the intraparietal area is crucial for both letter formation and letter imagery, but more extensive areas in the inferior parietal, superior occipital and precuneus gyri are involved in a severe deficit of letter imagery (letter recall). Also, in the intraparietal area, the anterior part seems to be more concerned with writing stroke sequences (described earlier).

Given these findings, we can now modify the dual-route hypothesis for writing that we described in Introduction. That is, the graphemic area (or graphic motor programs) in a narrow sense is located in the anterior intraparietal area posterior to the postcentral gyrus, stores visuokinesthetic [32] or sequential motor engrams for words and letters linked to handwriting, and sends this information to the frontal motor and premotor areas (hand area) (H in Fig. 6). The premotor hand area is located in the posterior middle and inferior frontal gyri (Areas 44/45 and 6), receives phonological, orthographic and visuokinesthetic information about words and letters from the posterior area, and links these three sources of information to send them to the motor area (Area 4) to execute handwriting. The orthographic route (b in Fig. 6) goes directly or indirectly via the parietal graphemic area (G in Fig. 6) to the frontal hand area. The direct orthographic route is used when a person recalls the visual image of a character, whereas the indirect orthographic route is used when he writes a character in response to dictation. The parietal graphemic area and frontal hand area have a reciprocal connection. As we write a word or letter repeatedly, visuokinesthetic and sequential motor information for words and letters is sent back from the frontal hand area to the parietal graphemic area, connects to the visual images from the orthographic route, and is stored there as an engram. This visuokinesthetic and sequential motor information is in turn transmitted to the frontal hand area when we write words spontaneously.

Patients with apraxic agraphia caused by damage to the graphemic area (G in Fig. 6) can spell aloud a word using the direct orthographic route, but probably cannot express the writing stroke sequence. Conversely, in disconnection type apraxic agraphia (interruption of output from G to H in Fig. 6) [29], patients not only can spell a word aloud but also can orally state the stroke sequence using the direct orthographic route and the recurrent route from H to G in Fig. 6. The interaction between the parietal graphemic area and frontal hand area also explains why a patient with letter shape imagery deficits can still produce well-formed letters [40]. According to the present theory, this patient had damage to the orthographic route at a point before the route is divided into the direct and indirect pathways. Moreover, "somesthetic dysgraphia" (patients 1 and 2) results from inappropriate sensory or kinesthetic feedback to the frontal hand area from the damaged postcentral somatosensory hand area.

This hypothesis predicts that apraxic agraphia caused by a lesion restricted to the anterior intraparietal area does not produce letter imagery deficits (impaired letter/character recall). This is because visual images of words and letters are transmitted through the intact subcortical association fibers via the direct orthographic route in the parietal lobe, although it is possible that the direct orthographic route is somewhat affected by the cortical damage. Unstable visual images of characters (patient 3) probably reflect this modulation effect of the deficient parietal cortex on the orthographic route or on the frontal hand area.

In addition, grapheme formation and letter recall are more disrupted when a lesion involves the posterior intraparietal, inferior parietal, superior occipital and precuneus gyri (patient 4). It is assumed that visuospatial attributes of a character (i.e. spatial alignment of each stroke) linked to handwriting are processed and stored there (graphemic area in a broad sense). As a result, kanji characters that have a complex configuration may be more deformed and difficult to recall than kana characters with this extensive lesion. Regarding this point, it should be noted again that activation of the superior parietal lobule and intraparietal area in both writing and mental recall was more widespread in kanji than in kana [26].

We should draw attention to the fact that letter/character imagery deficits (impaired character recall) do not solely occur because of damage to the parietal graphemic area, but also due to damage to the angular gyrus [14,15,17,25,38] and posterior inferior temporal cortex [19,36,41]. Our hypothesis can account for letter/character imagery deficits from different lesion sites, which are difficult to explain using the previous hypotheses.

As the present argument is based on only a few patients, further case studies are required to determine the anatomical substrates of grapheme deformity, writing stroke disorder and imagery deficits and also the role of the superior parietal lobule in writing.
